# School and Community-Based Interventions for Refugee and Asylum Seeking Children: A Systematic Review

**DOI:** 10.1371/journal.pone.0089359

**Published:** 2014-02-24

**Authors:** Rebecca A. Tyrer, Mina Fazel

**Affiliations:** Oxford University Department of Psychiatry, Warneford Hospital, Oxford, United Kingdom; University of California, San Francisco, United States of America

## Abstract

**Background:**

Research for effective psychological interventions for refugee and asylum-seeking children has intensified. The need for interventions in environments more easily accessed by children and families is especially relevant for newly arrived populations. This paper reviews the literature on school and community-based interventions aimed at reducing psychological disorders in refugee and asylum-seeking children.

**Methods and Findings:**

Comprehensive searches were conducted in seven databases and further information was obtained through searching reference lists, grey literature, and contacting experts in the field. Studies were included if they reported on the efficacy of a school or community-based mental health intervention for refugee or asylum-seeking children. Two independent reviewers made the final study selection, extracted data, and reached consensus on study quality. Results were summarized descriptively. The marked heterogeneity of studies excluded conducting a meta-analysis but study effect-sizes were calculated where possible. Twenty one studies met inclusion criteria for the review reporting on interventions for approximately 1800 refugee children. Fourteen studies were carried out in high-income countries in either a school (*n* = 11) or community (*n* = 3) setting and seven studies were carried out in refugee camps. Interventions were either primarily focused on the verbal processing of past experiences (*n* = 9), or on an array of creative art techniques (*n* = 7) and others used a combination of these interventions (*n* = 5). While both intervention types reported significant changes in symptomatology, effect sizes ranged from 0.31 to 0.93 and could mainly be calculated for interventions focusing on the verbal processing of past experiences.

**Conclusions:**

Only a small number of studies fulfilled inclusion criteria and the majority of these were in the school setting. The findings suggest that interventions delivered within the school setting can be successful in helping children overcome difficulties associated with forced migration.

## Introduction

The stressful experiences that many refugees and asylum-seekers are exposed to during forced migration, be that during persecution, flight and resettlement or in the changes they experience in their family, community and society make them vulnerable to a range of psychosocial problems [1]. As more is understood about the potential psychological sequelae of traumatic events experienced by refugees, research for effective interventions conducted in different settings has intensified [2]. These interventions can be delivered to individuals, families or groups and in either clinical or non-clinical/community settings. The intervention can either be focused on previous potentially traumatic events or can be multi-modal and comprehensive in design, concurrently addressing a number of issues in the child's environment and social networks as well as past experiences [3], [4]. The choice of potential interventions can therefore be limitless and so developing a coherent evidence-base is crucial to ensure that those interventions that are effective can be replicated and those that are not effective, discontinued.

The UNHCR estimated that at the end of 2012 there were 10.5 million refugees worldwide, of which approximately half were under the age of 18. Only a small proportion of all refugees reach high-income countries amounttagmk ting to less than half a million in 2011 [5]. A substantial proportion of those forcibly displaced from their homes move within their country of origin and are designated as internally displaced persons (IDPs) of which there were 17.7 million in 2012 [5]. Under the UN Refugee convention, the term ‘refugee’ is defined as someone who has fled their country of origin due to a well-founded fear of persecution because of race, religion, nationality, membership of a particular social group or political opinion [6]. An ‘asylum-seeker’ is waiting for their refugee status to be granted.

### Mental Health Issues in Refugee Populations

The prevalence of psychological disorders varies amongst refugees across studies, although high rates of post-traumatic stress disorder (PTSD) appears to be a common finding. A study which compared rates of psychological problems among 300 school children living in the UK showed that refugee children scored significantly higher than two control groups on the teacher-rated Strengths and Difficulties Questionnaire with one quarter of refugee children showing serious difficulties. The refugee children, when compared to non-refugee children from ethnic minorities and indigenous white children, had significantly more total difficulties (*p*<.01) [7]. Unaccompanied and separated children are often subject to increased risk not only of potentially traumatic events during their migration journey, but also of significant psychological difficulty after arrival [8]. In a recent study of war-exposed adult refugees resettled in Europe, high rates of mood disorders (43%), anxiety disorders (44%), and PTSD (33%) were reported. Stressful resettlement conditions were found to be significant contributing factors [9]. This suggests support is needed not only to tackle the traumatic events experienced pre-migration but also to address the on-going psychosocial stress in resettlement.

When refugee and asylum-seeking children arrive in a resettlement country, they might have experienced a host of potentially traumatic events depending on the conflicts they have left and the manner in which they have travelled to their new home. These experiences can be further confounded by post-migration events, such as stringent border controls, discrimination and social isolation which can raise the risk of developing psychological disorders [10], [11]. Furthermore, children have to negotiate a vast number of new challenges in a resettlement country such as learning a new language and understanding the educational and cultural environments of a new school. This process can be disrupted by changes in accommodation resulting in further school changes and low school attendance [12]. These stressors can be mirrored in their neighbourhoods and communities impacting on the natural resilience of families by further disrupting their environment. The post-migration environment, however, can play a crucial role in supporting refugee and asylum-seeking children and it is also an environment which is amenable to supportive interventions, such as those in the school or community-setting. It is for this reason that we conducted a systematic review to determine the evidence-base and possible effectiveness of such interventions.

### Refugee Camps

The 2011 UNHCR Global Report highlighted that one third of all refugees are living in camps or camp-like settings, with many likely to remain in them for several years [13]. Refugee camps present challenging living conditions where basic survival needs can become the overriding focus for families delaying restoration of the community and social mileau needed for healthy development [14]. It is estimated that vast numbers of children living in camps have significant psychological difficulties, exacerbated by the numerous adversities they can potentially experience, such as on-going insecurity, malnutrition, limited access to education, lack of work for parents, poor health and exposure to further violence and abuse [15]. Needless to say, mental health services in such settings are poorly available. There is a movement towards developing multimodal approaches to address mental and emotional health problems in these settings. For example, artistic activities in refugee camps have been used to engage recipients into ‘constructive action’ [14].

### The School Context for Mental Health Interventions

Schools could provide an ideal setting to implement interventions to address the mental health needs of refugee children. In disrupted environments, schools are often one of the earlier institutions to be introduced and, throughout the world, most children can attend school. Therefore the school is an environment that can potentially access children and their families. Schools can facilitate early identification and provide interventions to maximise cognitive, emotional and social development. Teachers and other school staff can identify children with difficulties as they observe children's behaviour in a range of settings, both structured and unstructured; over a long period of time and with different peers and adults [16]. School-based interventions delivered in a safe and informal setting potentially offer non-stigmatizing services which families may be more likely to accept given the increased likelihood of building relationships with school staff and the relatively easy access to children within school [17].

Birman *et al*., noted the school context is where the process of acculturation develops and therefore providing support either on an individual basis or using a multimodal approach may serve to enhance socialization and support psychological adjustment and development [18], [19]. Working with groups of children who have come together naturally in the school context can strengthen the child's relationship to the group through shared responsibilities, non-competitive activities and team work while simultaneously providing practical support [12].

### Drawing on the Literature

Investigation into successful mental health interventions for this population is warranted [1] as little is known about which theoretical models or implementation strategies are most appropriate [20]–[22]. Few programmes have been evaluated in the real-world setting of schools with even fewer designed for immigrant or refugee children [22]–[25]. Creative activities in the classroom that provide opportunities for children to construct personal accounts of their lives, interact with others and express emotion have consistently been found to have a beneficial effect on self-esteem, conflict resolution and problem solving [26], [27]. However, a literature review of interventions for refugee adults with PTSD and depression found trauma-focused cognitive behaviour therapy (TF-CBT) to be superior to other treatments [3]. A review of mental health interventions for children affected by war reported that creative-expressive, psycho-educational and recreational activities were most studied. Only a few studies had targeted specific PTSD symptomology using either TF-CBT or narrative exposure therapy (NET) [28]. This review was therefore conducted to systematically gather data on tested interventions to guide the development and understanding of the field.

### Aims of the Study

To conduct a systematic review of mental health interventions that had been evaluated in school or community-settings for refugee and asylum-seeking children.

## Methods

### Search Strategy

Seven databases were systematically searched: CINAHL; Embase; ERIC; PsycINFO; Scopus; Sociological Abstracts and Web of Science. Studies of mental health interventions in school and community-settings for asylum-seeking and refugee children reported from January 1987 to December 2012 were identified. The search was completed in January 2013. Searches of similar terms were combined such as “refugee”, “asylum-seeker”, “migrant”, “immigrant”, “displaced” with “school” “community” and “intervention” or “treatment”. The searches were limited to participants aged 2 to 17 years inclusive, and adaptations to the search terms were made in accordance with the requirements of each database. Additionally, grey literature was searched (WHO database), article reference lists and the authors of significant papers were checked for other relevant articles and experts in the field were consulted. There were no language restrictions.

### Criteria for Inclusion

Studies selected for inclusion were based on the following criteria:

1. Evaluation of a mental health intervention programme that addressed emotional, social or behavioural difficulties of the sample using a controlled or within-subjects experimental design
2. The population was inclusive of IDPs, asylum-seekers and refugees
3. Target age: 2 to 17 years inclusive
4. Intervention delivered in schools, refugee camps or the community as opposed to clinic and hospital-based settings
5. Intervention outcome was evaluated with a clinical psychometric measure

### Studies for exclusion

Studies selected for exclusion were Interventions that:

1. Evaluated educational performance or language acquisition
2. Aimed to change the overall school environment without specific measures taken on the asylum-seeking and refugee children
3. Evaluated non-displaced children and adolescents in areas of on-going conflict
4. Reported single case studies

### Quality of Ratings Scale

Following a broad review of quality rating scales [29] the Yates Scale was chosen to evaluate the quality of the studies as it was comprehensive and has been used in similar reviews [30]. As a quality rating scale it has face, content and construct validity with good reliability however, its criterion validity and internal consistency are not strong [29]. The Yates scale focuses on the quality of two main areas: Quality of design and methods and Treatment quality. The quality rating of each study was assessed independently by each author (RT and MF) and any discrepancies in results discussed.

In the Yates Scale, the evaluation of quality of design and methods includes questions on study sampling, minimisation of bias, outcome measures, control groups and statistical analyses. Scores range from 0 to 26 and cut-offs determined in another study were used (0–8: ‘not fulfilled’; 9–17: ‘partially fulfilled’; 18–26: ‘fulfilled’) [31]. The evaluation of treatment quality includes questions on the rationale and explanation of the treatment, whether it is manualised, therapist training and patient engagement. Scores range from 0 to 9 (0–3: ‘not fulfilled’; 4–6: ‘partially fulfilled’; 7–9: ‘fulfilled’).

### Effect Size

Effect sizes of the study interventions were either obtained from the publications when provided or calculated for this review using a procedure outlined by Thalheimer and Cook [32] and cross-checked against a web-based calculator [33]. Cohen's *d* effect sizes were computed for symptom change to try and present data in a manner that could be compared across studies, given the high clinical heterogeneity of the sample [34]. The calculations were conducted using the average standard deviations between two means, therefore calculations could only be conducted for studies with a control group. Cohen proposed *d* = 0.2 as a small effect size, *d* = 0.5 as a moderate effect size, and *d* = 0.8 as a large effect size [34]. As limited follow-up data were available, the effect sizes were calculated from end of treatment scores.

## Results

**Figure 1:**
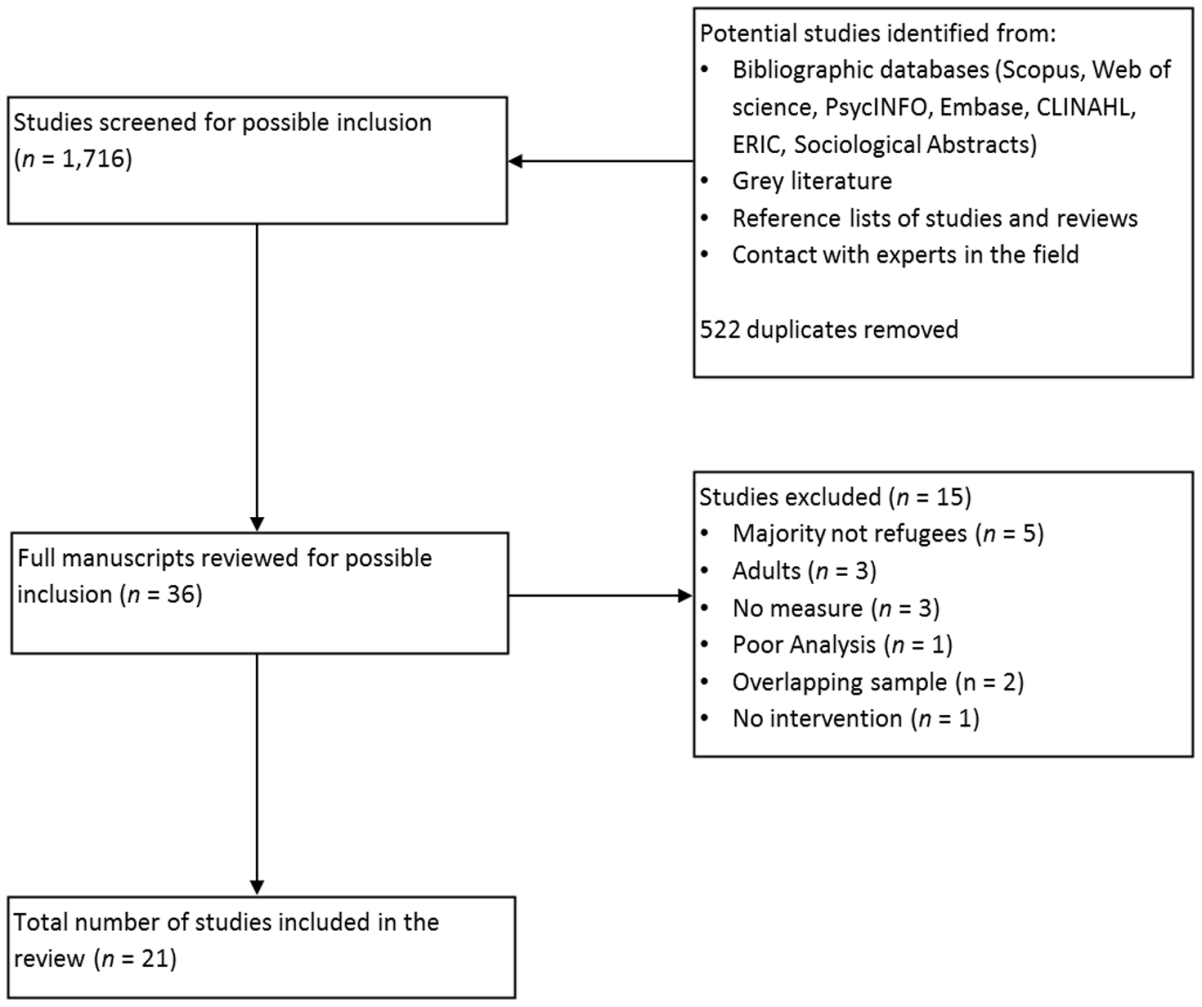
Flow Diagram to show the process of Study Selection.

The database search identified 2,237 potentially relevant papers, of which over 500 were duplicates and the majority were not describing an intervention. 36 full papers were reviewed of which 23 met inclusion criteria reporting on 21 studies (refer to Figure 1 for the process of study selection). Two online publications were subsequently published in print [35], [36]. The majority of papers were excluded on initial screening because they did not report on an intervention or the intervention reported was conducted on adults, non-refugee populations, or in hospital settings. Two papers reported on subsamples of larger included studies [37], [38]. The studies were undertaken in ten different countries on either specific refugee populations or mixed groups of migrant children, including refugees. Four authors provided further information on their studies (A. Ager, personal communication, November, 24, 2013; D. Birman, personal communication, December, 24, 2012; M. Hodes, personal communication, May, 20, 2013 & E. Newnham, personal communication, May, 20, 2013). Through searching article reference lists one unpublished study was identified which could not be obtained [39].

### Intervention features

All twenty one studies meeting inclusion criteria were published since 2000 and included data from approximately 1,800 children (some studies included other migrant children). These reported school and community-based interventions aimed at the mental health, psychosocial development and functioning of asylum-seeking and refugee children. Table 1 presents a summary of the studies included with information on the intervention used, the population targeted and the assessment of study quality. Given the marked difference of refugee camp settings, the interventions that were provided in these camps are presented separately.

**Table 1:** Summary of included studies.

First Author	Setting	Country	Intervention focus	Intervention	Instrument used	Study type	Target population	Selection criteria	Sample size*	Age yrs	Quality of design and methods	Treatment quality
**STUDIES from High-income settings**												
**Baker 2006** [52]	School	Australia	Creative arts (music therapy)	Group	BASC	CCT: Cross-over design	Newly arrived refugees	Present at school for following two terms	31(31)	11–16	Partially fulfilled	Not fulfilled
**Barrett 2003** [56]	School	Australia	CBT (FRIENDS)	Group	SEI, RSES, RCMAS, TSCL, BHS, KHS	Case-control study: classes grouped together	Mixed migrant population, approx. half refugees	ESL class	166	6–19	Partially fulfilled	Fulfilled
**Beehler 2012** [17]	School	USA	CBT, TF-CBT, comprehensive (CATS)	Individual, Group	CAFAS, PTSD-RI	Cohort study: two school districts	Mixed migrant population, small proportion refugees	Referred by staff, nurses or parents	149	6–21	Not fulfilled	Partially fulfilled
**Birman 2008** [40]	Community	USA	Comprehensive (FACES), Counselling, therapy, creative arts	Individual, Family & Group	CAFAS, HTQ	Cohort study: attending specialist service	57% Refugees and asylum seekers, 43% other types of migrant	Needing further intervention	97 (68)	6–18	Not fulfilled	Not fulfilled
**Dura-Vila 2013** [36]	School	UK	Individual, family & supportive therapy.	Individual, Family	SDQ	Cohort study: referred to specialist service	Refugees and asylum seekers	Referred by teachers & social workers	92 (74)	3–17	Not fulfilled	Not fulfilled
**Ehntholt 2005** [12]	School	UK	CBT	Group	R-IES, DSRS, RCMAS, WTQ, SDQ	CCT	Asylum-seekers	Referred by teachers	15	11–15	Not fulfilled	Fulfilled
**Ellis 2013** [35]	Community	USA	Comprehensive, Skill-based groups +/-TST	Individual, Group	WTSS, PWA,PTSD-RI, DSRS	Cohort study: attending a school	Somali refugees	All Somali ESL children	30 (26)	11–15	Fulfilled	Partially fulfilled
**Fazel 2009** [41]	School	UK	Supportive therapy & creative arts	Individual, Family & Group	SDQ	Cohort study: referred to specialist service	Refugees and asylum seekers	Referred by teachers	69 (47)	5–17	Partially fulfilled	Not fulfilled
**Fox 2005** [54]	School	USA	CBT	Group	CDI	Cohort Study	South-East Asian refugees	All those attending a school	58	6–15	Not fulfilled	Not fulfilled
**Kalantari 2012** [44]	School	Iran	Exposure through writing	Group	TGIC	RCT	Afghan refugees	High score on traumatic grief measure	29 (29)	12–18	Partially fulfilled	Partially fulfilled
**Möhlen 2005** [55]	Community	Germany	Trauma-focus therapy and Creative arts	Individual, Family & Group	HTQ, K-SADS, DYSIPS, CGAS	Cohort study	Kosovo-Albanian refugees	In refugee accommodation.	10 (10)	10–16	Not fulfilled	Partially fulfilled
**Rousseau 2005** [45]	School	Canada	Creative arts (CEW)	Group	TRF, CSCS, Dominic Interactive	RCT: whole classes randomly assigned	Mixed migrant, mainly Asian & South American	Students in special integration and normal classes	73 (73)	7–13	Partially fulfilled	Not fulfilled
**Rousseau 2009** [46]	School	Canada	Creative arts (CEW-sandplay)	Group	SDQ	RCT: whole classes randomly assigned	Predominantly South Asian (28% refugees)	All students	52	4–6	Fulfilled	Partially fulfilled
**Schottelkorb 2012** [47]	School	USA	TF-CBT vs. creative arts (CCPT)	Individual vs. group	UPID, PRPS	RCT	Refugees	Referred by teachers	31 (26)	6–13	Partially fulfilled	Fulfilled
**STUDIES from REFUGEE and IDP CAMPS**												
**Ager 2011** [48]	School in IDP camp area	Uganda	Creative arts (PSSA)	Classroom	Modified BEI	RCT: schools randomly assigned	Ugandan IDPs	Referred by teachers	203 (191)	7–12	Partially fulfilled	Partially fulfilled
**Bolton 2007** [49]	Camp	Uganda	IPT vs. creative arts (CP)	Group	APAI	RCT	Ugandan IDPs	High score on depression scale	210 (210)	14–17	Partially fulfilled	Fulfilled
**Catani 2009** [50]	Camp	Sri Lanka	KIDNET vs. meditation relaxation	Individual vs. group	UPID, authors' functioning scale	RCT	Sri Lankan IDPs	Children in new camps with preliminary PTSD diagnosis	31 (31)	8–14	Partially fulfilled	Fulfilled
**Ertl 2011** [51]	Camps	Uganda	NET vs. academic catch-up and counselling	Individual	CAPS, MINI, VWAES, adapted stigma scale	RCT	Ugandan former child soldiers	PTSD diagnosis	57 (57)	12–25	Fulfilled	Fulfilled
**Gupta 2008** [43]	Camp	Sierra Leone	Creative arts (Rapid-Ed)	Group	IES	Cohort study	Sierra Leonean IDPs	Randomly selected from school registration lists	315 (306)	8–17	Not fulfilled	Partially fulfilled
**Onyut 2005** [42]	Camp	Uganda	KIDNET	Individual	PDS, HSCL, CIDI	Cohort study (pilot)	Somali	PTSD diagnosis	6	13–17	Partially fulfilled	Fulfilled
**Thabet 2005** [53]	Schools in camp area	Gaza	Creative arts (modified CISM) vs. teacher psycho-education	Group	CPTSD-RI, CDI	CCT	Palestinians residing in camps	High PTSD symptom scores	69	9–15	Fulfilled	Partially fulfilled

*Sample size calculated excluding non-active controls; brackets indicate final number used in evaluation, if reported.

APAI: Acholi Psychosocial Assessment Instrument; BASC: Behaviour Assessment System for Children; BEI: Brief Ethnographic Interviewing; BHS: Beck Hopelessness Scale; CAFAS: Child and Adolescent Functional Assessment Scale; CAPS: Clinical-Administered PTSD Scale; CATS: Cultural Adjustment and Trauma Services; CBT: Cognitive Behaviour Therapy; CCPT: Child-Centered Play Therapy; CCT: Controlled Clinical Trial; CDI: Children's Depression Inventory; CEW: Creative Expression Workshops; CGAS: Child Global Assessment Scale; CIDI: Composite International Diagnostic Interview; CISM: Critical Incident Stress Management; CP: Creative Play as developed by War Child Holland; CPTSD-RI: Child Post Traumatic Stress Reaction Index; CSCS: Piers-Harris Children's Self-Concept Scale; DSRS: Depression Self-Rating Scale; DYSIPS: Diagnostic Symptom for Psychological Disorders; ESL: English as a Second Language; FACES: Family, Adult and Child Enhancement Services; HSCL: Hopkins Symptom Checklist-25;HTQ: Harvard Trauma Questionnaire; IDP: Internally displaced person; IES: Impact of Events Scale; IPT: Interpersonal therapy; KHS: Kazdin Hopelessness Scale; KIDNET: Narrative Exposure Therapy adapted for children; K-SADS: Kids Schedule for Affective Disorders and Schizophrenia; MINI: Mini International Neuropsychiatric Interview; NET: Narrative Exposure Therapy; PDS: Posttraumatic Diagnostic Scale; PRPS: Parent Report of Posttraumatic Symptoms; PSSA: Psychosocial Structured Activities Program; PTSD-RI: PTSD Reaction Index; PWA: Adolescent Post-War Adversities Scale-Somali Version; RCMAS: Revised Children's Manifest Anxiety Scale; RCT: Randomised Clinical Trial; R-IES: Revised Impact of Events Scale; RSET: Rosenberg Self-Esteem Scale; SEI: Self-Esteem Inventory; SDQ: Strengths and Difficulties Questionnaire; TF-CBT: Trauma-Focused Cognitive Behaviour Therapy; TGIC: Trauma Grief Inventory for Children; TRF: Achenbach's Teacher's Report Form; TSCC: Trauma Symptom Checklist for Children; TSCL: Trauma Symptom Checklist for Children; TST: Trauma Systems Therapy; UPID: UCLA PTSD Index for DSM-IV;WTQ: VWAES: Violence, War and Abduction Exposure Scale; War Trauma Questionnaire; WTSS: War Trauma Screening Scale.

**Figure 2:**
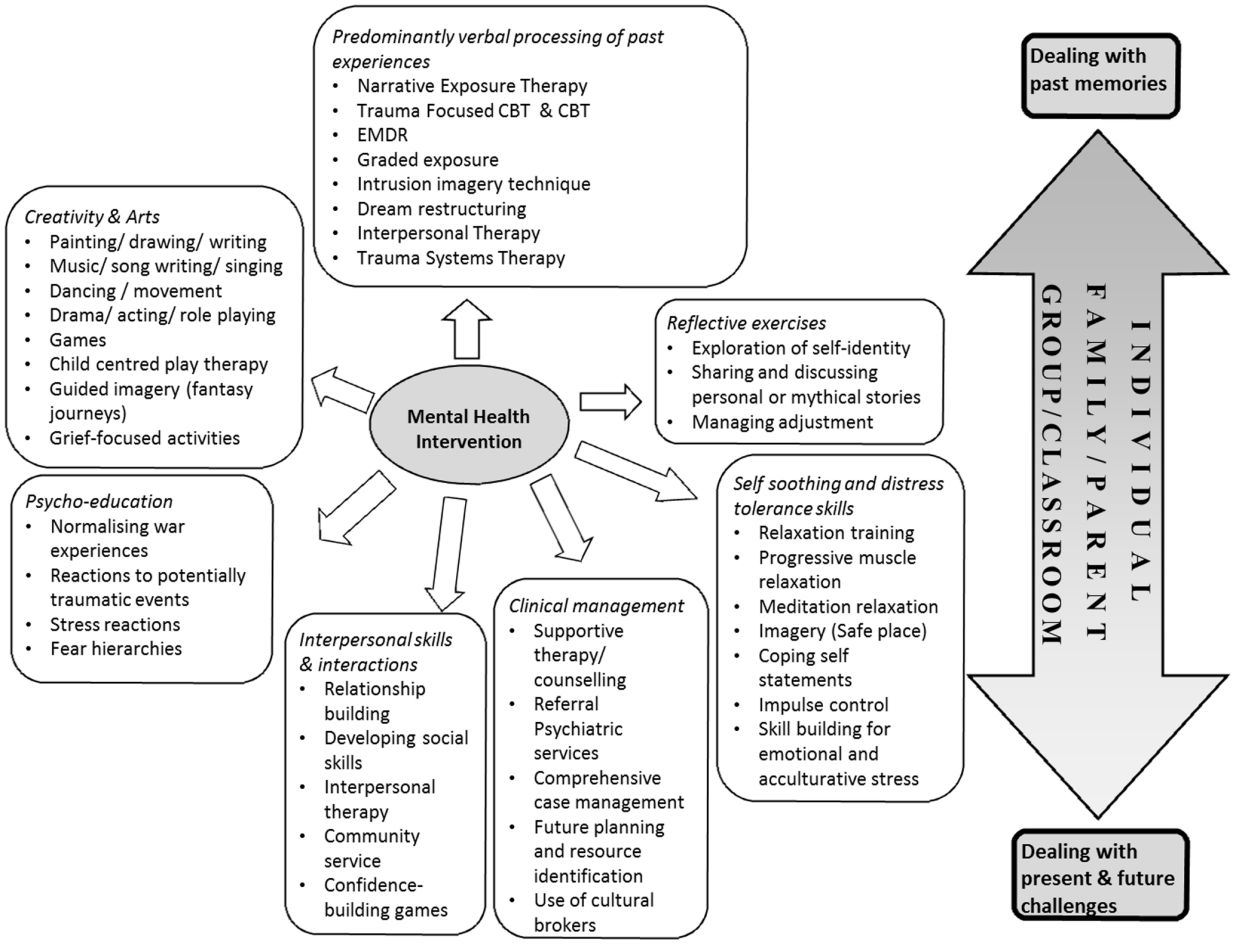
Diagram to show the range of mental health interventions included in the selected studies.

Due to the considerable variation in the types of intervention being delivered and the populations targeted by each intervention, a meta-analysis could not be conducted due to the significant clinical heterogeneity of the samples. Two broad classes of intervention were identified, firstly interventions based primarily on the verbal processing of past experiences (*n* = 9), and secondly, creative art techniques (*n* = 7) with five further studies using a combination of both. The verbal processing approaches included CBT and TF-CBT; NET, Eye-Movement Desensitization and Reprocessing (EMDR) and Trauma Systems Therapy (TST). The creative art techniques drew on an array of different therapies including music therapy, creative play, drama and drawing. The range of different mental health interventions utilised in the included studies is shown in Figure 2. Services were delivered either in the school (*n* = 11), community (*n* = 3) or refugee camps (*n* = 7 of which 2 were in camp schools). Of these, four studies included consultation meetings with professionals working in other agencies [35], [36], [40], [41].

### Study Quality

#### Quality of design and methods

In assessing overall quality of design and methods in the studies, four studies scored ‘fulfilled’; ten ‘partially fulfilled’ and seven ‘not fulfilled’. For example, information on attrition rates (participants lost at follow-up) was only reported in six studies and minimising biases reported in 18 studies (four ‘fulfilling’ criteria and 14 partially fulfilling criteria). All 21 studies fulfilled the criteria for statistical reporting.

The sample sizes of included studies ranged from 6 participants [42] to 315 participants [43]. Eight studies used random allocation to determine groups [44]–[51]. Four studies were controlled clinical trials [12], [35], [52], [53], eight were cohort designs [17], [36], [40]–[43], [54], [55] and one was a case control study [56]. Recruitment strategies differed across studies; in six studies children were selected to receive an intervention based on meeting specific criteria [42], [44], [49]–[51], [53]. In five studies a whole class received the intervention [35], [45], [46], [54], [56]. Seven studies used referrals from school staff [12], [17], [36], [40], [41], [47], [48]. In three studies children were either selected on the basis of their refugee status [52], their residence in refugee accommodation [55], or randomly selected from a school register [43].

#### Treatment quality

In assessing treatment quality, seven studies scored ‘fulfilled’, eight ‘partially fulfilled’ and six ‘not fulfilled’. Interventions typically lasted 10–12 weeks although there was a range from a fortnight [50] to 16 weeks [49]. The number of sessions varied between 6 and 17, most commonly lasting one hour. In three studies, interventions were conducted over the course of a school year [35], [36], [41]. A further two studies enlisted a range of individual and group therapies and longitudinal data were collected and analysed [17], [40]. Beehler *et al*., collected data over a 3 year period (the number of sessions cannot be inferred) [17], and Birman *et al*., engaged participants in services for 1 month to 7 years [40]. Parents were involved in six interventions [17], . Three studies involved family therapy sessions [17], [36], [40], one involved individual parental support [48] and two studies incorporated both family sessions and individual parental sessions [41], [55]. In one study, school staff also received weekly consultation with mental health professionals at the schools [41].

Two studies, both fulfilling most of the quality criteria, are described in the text boxes. Text Box 1 describes a CBT-based intervention in schools [56] and Text Box 2 describes a NET trial conducted in a refugee camp [51].

### Effectiveness of the interventions

The intervention programmes reviewed addressed a range of difficulties experienced by asylum-seeking and refugee children. The studies reporting significant changes in psychological symptoms are summarised in Table 2. Cohen's *d* effect sizes are reported for the seven studies that provided sufficient data for these to be calculated, five of which were for therapies based on verbal processing of previous traumatic events [12], [44], [49], [51], [56]. The effect sizes ranged from 0.31 to 0.93 and six of the studies had effect sizes in the medium to large range.

**Table 2:** Summary of significant findings in studies.

First author	Intervention	Significance	Effect size Cohen's *d* (data permitting)
**Depression**			
Barrett, 2003[56]	CBT	Decrease in hopelessness symptoms in high school students as measured by the BHS (*p*<.01)	0.93
*Bolton, 2007* [*49*]	*IPT*	*Group IPT reduced depressive symptoms (p = .02)*	*0.57*
Ellis, 2013[35]	TST	Decrease in depression symptoms (p = .011) as measured by the DSRS	
Fox, 2005[54]	CBT	CBT reduced depressive symptoms (*p*<.001)	
Möhlen, 2005[55]	Creative arts	Range of creative art techniques reduced depressive symptoms (*p* = .014)	
**Anxiety**			
Barrett, 2003[56]	CBT	Anxiety symptoms decreased following group based CBT for elementary school (*p*<.001) and high school students (*p*<.05) as measured by the RCMAS	0.93 (elementary)0.67 (high)
Ehntholt, 2005[12]	CBT	Decrease in anxiety symptoms (p = .018) as measured by the RCMAS	0.64
Möhlen, 2005[55]	Creative arts	Range of creative art techniques reduced anxiety symptoms (*p* = .006)	
**PTSD**			
Barrett, 2003[56]	CBT	Decrease in PTSD symptoms for high school students with group based CBT (*p*<.001) on the TSCC PTSD subscale	0.92
Beehler, 2012[17]	CBT, TF-CBT, comprehensive	Decrease in PTSD symptoms with TF-CBT (*p*<.05), supportive therapy (*p*<.04) and a decreasing trend was found with CBT (*p*<.07).	
*Catani, 2009* [*50*]	*KIDNET & meditation-relaxation*	*NET and meditation-relaxation reduced PTSD symptoms, sustained at follow-up (p<.001)*	
Ehntholt, 2005[12]	CBT	Decrease in PTSD symptoms (*p* = .011) as measured by the IES	0.88
Ellis, 2013[35]	TST	Decrease in PTSD symptoms (p = .016) as measured by the PTSD-RI	
Ertl, 2011[51]	NET	Decrease in PTSD symptoms with NET, as measured by the CAPS, compared to supportive counselling (*p* = .02) and waiting list controls (*p* = .02)	0.31
*Gupta, 2008* [*43*]	*Creative arts: Rapid-Ed*	*Decrease in PTSD symptoms in 96% of participants following intervention.*	
Möhlen, 2005[55]	Creative arts	Decrease in PTSD symptoms with a range of creative art techniques (*p* = .018)	
*Onyut, 2005* [*42*]	*KIDNET*	*Decrease in PTSD symptoms with KIDNET (p = 0.039)*	
Schottelkorb 2012 [47]	TF-CBT & CCPT	Both interventions significantly decreased PTSD symptoms in those with symptom scores in the clinical range (child and parent-reported measures)	
**Functional impairment**			
Beehler, 2012[17]	CBT, TF-CBT, & supportive therapy	Decrease in functional impairment with TF-CBT (*p*<.01), supportive therapy (*p*<.001) and CBT (*p*<.03).	
Birman, 2008[40]	Comprehensive service, counselling, therapy, creative arts	Decrease in functional impairment following a mixed intervention of cognitive therapy and creative arts (*p*<.001).	
*Catani, 2009* [*50*]	*KIDNET & meditation-relaxation*	*Decrease in functional impairment sustained at follow-up with both KIDNET and meditation-relaxation (p<.001).*	
*Ertl, 2011* [*51*]	*NET*	*Decrease in functional impairment with NET compared to supportive counselling (p = .008) and waiting list controls (p<.001)*	*0.64*
**Other**			
Barrett, 2003[56]	CBT	Anger: Decrease in levels of anger (*p*<.001) in high school students as measured by the TSCC Anger scale	0.79
Ehntholt, 2005[12]	CBT	Behavioural problems: Decrease in behavioural problems (*p* = .027) as measured by the SDQ	
Ehntholt, 2005[12]	CBT	Emotional problems: Decrease in emotional problems (*p* = .010) as measured by the SDQ	0.32
Rousseau, 2009[46]	Sandplay	Emotional problems: Decrease in parent-rated SDQ emotional problems (*p* = .002)Relational problems: Decrease in parent-rated relational problems (*p* = .001)	0.430.48
Ellis, 2013[35]	TST	Resource hardship: Decrease in resource hardship (p = .027)	
Durà-Vilà, 2013[36]	Individual, family & supportive therapy	Conduct problems: Decrease in parent-rated conduct problems (*p* = 0.043)Hyperactivity: Decrease in teacher-rated (p = 0.015) and parent-rated (p = 0.001) hyperactivityPeer Problems: Decrease in teacher-rated peer problems (p = 0.017) as measured by the SDQ	
Fazel, 2009[41]	Supportive therapy & creative arts	Peer Problems: Decrease in teacher-rated peer problems for both CBT and creative arts therapy (*p* = .005)	
Kalantari, 2012[44]	Exposure through writing	Traumatic grief: Decrease in children's traumatic grief symptoms (*p*<.001) as measured by the TGIC	0.67
Rousseau, 2005 [45]	Creative expression	Mental health symptoms: Decrease in self-reported mental health symptoms	
*Ager, 2011* [*48*]	*Creative arts*	*Well-being: Improved well-being at 12 months according to self-rated (p<.001), and parent-rated (p = .01) measures but not teacher ratings (p>.1)*	*0.75 (self-rated); 0.5 (parent-rated)*

Italicized studies indicate those conducted in refugee and IDP camps.

CAPS: Clinical-Administered PTSD Scale; CBT: Cognitive Behaviour Therapy; CCPT: Child-Centered Play Therapy; IPT: Interpersonal therapy; KIDNET: Narrative Exposure Therapy adapted for children; NET: Narrative Exposure Therapy; PSSA: Psychosocial Structured Activities program; PTSD-RI: PTSD Reaction Index; RCMAS: Revised Children's Manifest Anxiety Scale; SDQ: Strengths and Difficulties Questionnaire; TF-CBT: Trauma-Focused Cognitive Behaviour Therapy; TGIC: Trauma Grief Inventory for Recovery; TSCC: Trauma Symptom Checklist for Children; TST: Trauma Systems Therapy.

Both the verbal processing-based and creative art-based interventions led to significant reductions in symptoms of depression, anxiety, PTSD, functional impairment and peer problems. Verbal processing therapies were also effective in treating anger [56], traumatic grief [44], resource hardship [35], behavioural and emotional problems [12], and hyperactivity, peer and conduct problems [36]. Creative arts were also effective in treating well-being [48], and emotional and relational problems [46].

All but one study conducted in refugee and IDP camps found significant findings [53]. Two of these studies reported a significant decrease in functional impairment following NET [50], [51]. Two studies found a decrease in PTSD symptoms following a creative arts intervention [43] and KIDNET (an adapted version of NET for children and adolescents) [42]. Bolton found interpersonal therapy (IPT) reduced symptoms of depression [49] and Ager found improvements in well-being following a psychosocial activities programme [48].

Five studies reported significant reductions in symptoms of depression [35], [49], [54]–[56]. Two of these studies used CBT. Bolton found IPT superior to an activity-based intervention in treating symptoms of depression (*p* = .02). Furthermore, the activity-based intervention was no more effective than waiting list controls in treating depression [49]. Although these results point towards the importance of the cognitive behavioural approach in treating depression in refugee children it should be noted that Möhlen found a range of creative art techniques significantly reduced symptoms of depression (*p* = .014) [55].

Three studies reported a significant improvement in symptoms of anxiety. Group-based CBT and a creative art-based intervention incorporating psycho-education, creative techniques and relaxation activities in individual, family and group sessions were found to decrease levels of anxiety [12], [55], [56].

Nine studies reported a decrease in symptoms of PTSD among asylum-seeking and refugee children [12], [17], [35], [42], [43], [50], [51], [55], [56]. All but one of these treatments was grounded in the verbal processing of past experiences. Four of the studies were undertaken in low-income countries [42], [43], [50], [51].

Only four studies reported improvements in functional impairment [17], [40], [50], [51] incorporating a range of interventions. Catani found no significant difference in functional impairment following KIDNET or meditation-relaxation although at six month follow up recovery rates for KIDNET were higher at 81% as opposed to 71% [50]. Ertl, however, found functional impairment improved significantly with NET compared to supportive counselling (*p* = .008) and waiting list controls (*P*<.001) [51]. In the Birman study, participants received tailored services to meet their individual needs; it is therefore difficult to evaluate which elements of the intervention had the greatest impact on improvements in functioning [40]. Similarly, Beehler utilised a variety of interventions including TF-CBT, supportive counselling and other CBT approaches [17].

## Discussion

Despite millions of children affected by forced migration only limited evidence is available as to possible school and community interventions to support the mental health of this group. Overall, 21 studies were identified, most conducted in schools with a variety of therapeutic tools and modalities utilised. Of the eight studies from LMICs, seven were conducted in refugee camps. Many of the interventions focused on past traumatic events, either using verbal processing, for which there is the strongest evidence-base, or by using an array of creative arts techniques. Significant improvements were seen for depression, anxiety, PTSD, functional disturbances and peer problems in both types of interventions. Individual as well as group interventions were effective; as were both short and long-term treatments. CBT or TF-CBT and NET both have evidence to support their use. Some services developed comprehensive interventions. Effect sizes calculated to compute symptom change in disorders were, however, primarily available for interventions based on the verbal processing of past experiences.

Six out of the seven studies conducted in refugee camp settings showed a significant reduction in psychological symptoms. The success of these interventions are noteworthy given that one third of all refugees will spend some time in a refugee camp, either in their own country or a neighbouring low and middle-income country (LMIC) [13]. NET was used in three of these studies and is an example of how complex interventions can be delivered in resource-poor settings.

Recent studies have highlighted the importance of offering comprehensive or multi-modal services to refugees and their families. Multimodal interventions aim to concurrently address issues of psychological functioning, social and cultural adaptation, physical health and ongoing psychosocial difficulties [3]. These multimodal interventions are thus integrated into other systems of care, such as women's health or primary care and might play a particularly important role in contexts where mental health resources are scarce. Although the evidence supporting their use is limited, these services try to address the complex array of difficulties refugee and asylum seeking families might encounter. At the societal level, they might try to influence the wider environment through advocating for more services and stable housing, promoting language proficiency, improving immigration applications and employment opportunities. The restoration of a supportive environment for the young person and their family is likely to be key to stabilising their psychological health [28], [57]. This ensures that all the needs identified by the individual or family are addressed and the focus is not entirely on their mental health [4], [58]. Restoration of social support networks for children and their families is another important aspect of multimodal interventions and have been demonstrated in post-conflict settings [59]. The importance of harnessing cultural resources and extended kin networks are likely to also be important [60] and some of the studies included in this review included ‘cultural brokers’ in the mental health teams. In this systematic review, only three of the included studies were comprehensive and multimodal in design [17], [35], [40].

These studies therefore highlight the importance of addressing previous traumatic experiences utilising approaches that focus on exposure to the event in question through verbal processing. The studies that used CBT had the largest effect sizes. The evidence supporting the many different creative arts techniques is, at present, not as robust, however, interesting evidence is emerging in both post-conflict and post-migration environments on the importance of multimodal treatments. Many questions regarding treatments, therefore, remain unanswered and warrant further exploration. Collecting variables on educational attendance and attainment, future aspirations of individuals and the overall school climate from the perspective of students and staff would be important to determine the impact of services located within schools. Only five of the 21 included studies had more than 100 participants and so larger controlled studies with longitudinal collection of data will provide much needed evidence of effectiveness. Studies could elucidate the differential impact of effective treatments, such as a comparison of TF-CBT and NET; or determine whom to include in treatment by exploring interventions incorporating families, peers and school staff; as well as exploring the influence of different community locations for treatment such as working within the school compared to a local health clinic or within the family home. Answering some of these questions could enable a better appreciation of factors influencing therapeutic effectiveness, acceptability of treatment and engagement for these populations who are difficult to access in traditional services.

### Limitations

Several limitations of this review should be highlighted. Of the 21 studies included, only eight monitored treatment fidelity and eight conducted a follow-up assessment. In combination with small sample sizes, lack of blind assessment, and inactive or no control groups the overall quality of studies reviewed was a limitation and highlights the areas needed to be addressed by further work. Participant eligibility varied across studies; in the majority of cases refugees and asylum-seekers were enrolled in treatment irrespective of whether they met clinically significant rates of psychological problems prior to the commencement of the intervention.

The studies were varied in their scope, environment and target population and so limited conclusions can be drawn on what is most effective for these settings. The interventions adopting the most multimodal approaches attempting to address both systemic and individual needs were those with the lower quality ratings. This could reflect the difficulty of evaluating more complex interventions trying to address potential difficulties in community, school and refugee camp settings [17], [36], [40].

Studies of interventions for children living within current conflict conditions were excluded but could have provided some important examples of interventions. There have, however, been two recent comprehensive systematic reviews on mental health interventions for children living in conflict and post-conflict environments [28], [57].

## Conclusion

Refugee children arriving in a new country, either with or without their families, are likely to benefit from schools and services that can enable them to settle in their new environment. For those arriving in high-income countries, for example, accessing services can be fraught with difficulties due to linguistic, social, and historical reasons [61]. Cultural and family beliefs about psychological difficulties can also prevent parents or carers seeking professional help [37]. Furthermore, caregivers might not recognise some difficulties in children as being a manifestation of psychological problems. Past experiences faced by refugees can also make it difficult to establish a sense of trust necessary for a therapeutic relationship [62]. As a result, mental health services can experience difficulties in reaching these children and it is therefore important to determine the value of offering services in other settings [60], [63]-[66]. These problems are overshadowed by the many larger difficulties faced in providing services in LMICs [1], however, some studies included in the review have been able to demonstrate impressive results in low-resource settings.

Adolescents derive psychological benefit from feeling they belong to a school yet this task can be particularly difficult if one arrives with limited knowledge of the local language and culture. This can be further complicated if they are more conspicuous at school and bullied as a result [67]. Schools are often recommended as a location for interventions because they can be familiar, non-stigmatising environments that offer broad access to children and families [20], [59], [68]. Developing sustainable and accessible interventions are essential and training local non-mental health professionals to deliver interventions could address this need [69]. Some studies utilised lay therapists successfully, a model that needs replication in other settings and with other therapeutic modalities. To this end, within schools, teachers or other members of school staff could be trained to promote mental health by creating a supportive and caring environment and through implementation of preventative and efficacious psychological interventions [12].

Parents and other primary caregivers can be compromised in the context of societal violence and subsequent migration and therefore families need to be supported in the community [60], [63]–[65]. Interventions to try and address the overall environment of refugee children are therefore important, not only for unaccompanied minors [8] but all refugee children attending schools and living in new places. Longitudinal studies underline the importance of addressing these issues, as a study of refugees two decades after settlement in America showed how persistent later psychological problems are, especially if the refugees are unemployed and living in poverty [66].

The different contextual factors, environments and socio-cultural political contexts that refugees come from and find themselves in cannot be ignored [70] and services need to try and address the heterogeneity of difficulties, both past and present, that refugees experience [3]. This is the rationale for offering a broad range of services to refugee children [36] yet the evidence-base remains weak to support this approach over individualised trauma-focused work. For adult refugee populations and other traumatised children, CBT is the most studied and effective intervention [3], [71]. There is, however, probably a need to also address current daily stressors [4] although interventions should not undermine natural recovery processes [69].

Achieving in school, with regards to both education and peer relationships, is a key determinant of success and future mental health [72]. War and conflict disrupt social, educational and economic systems and these exert effects on psychological well-being in complex ways [4]. These disruptions disproportionately affect the young and their transitions into adult life [72]. In particular, for younger populations the importance of family, peer and educational domains are crucial to help them fulfil their potential [72] and examples of effective mental health interventions are highlighted in this review.

## Supporting Information

Diagram S1 (DOC)

Checklist S1 (DOC)

Text Box S1 **FRIENDS programme delivered in a school setting.**
(TIF)

Text Box S2 **Narrative Exposure Therapy delivered in refugee camp setting.**
(TIF)
