# Correction: School and Community-Based Interventions for Refugee and Asylum Seeking Children: A Systematic Review

**DOI:** 10.1371/journal.pone.0097977

**Published:** 2014-05-12

**Authors:** The PLOS ONE Staff

## Notice of Republication

This article was republished on April 25th, 2014, due to figures and tables being incorrectly left out of the article or duplicated in the Supporting Information. The publisher apologizes for this error. Please download the PDF again to view the corrected article. The originally published, uncorrected article and the republished, corrected article are provided here for reference.

## Supporting Information

File S1 Originally published, uncorrected article.
(PDF)

File S2 Republished corrected article.
(PDF)
