# Supplementary material for: School and Community-Based Interventions for Refugee and Asylum Seeking Children: A Systematic Review
Source: PLoS One. 2014 Feb 24;9(2):e89359. doi: 10.1371/journal.pone.0089359 (PMC3933416; doi:10.1371/journal.pone.0089359)
Supplement: Diagram S1 — (DOC) [file pone.0089359.s001.doc]

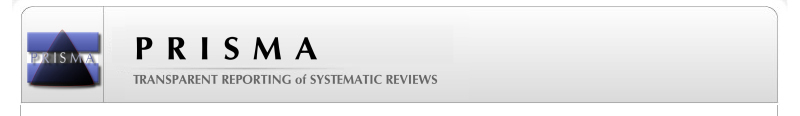
**PRISMA 2009 Flow Diagram**

**Screening**

**Included**

**Eligibility**

**Identification**

Records identified through database searching
(n = 2,236)

Additional records identified through other sources
(n = 2)

Records after duplicates removed
(n = 1,716)

Records screened
(n = 1,716 )

Records excluded
(n = 1,680)

Full-text articles assessed for eligibility
(n = 36)

Full-text articles excluded, with reasons
(n = 15)

Studies included in qualitative synthesis
(n = 21)

Studies included in quantitative synthesis (meta-analysis)
(N/A)
