# Supplementary figures and images for: School and Community-Based Interventions for Refugee and Asylum Seeking Children: A Systematic Review
Source: PLoS One. 2014 Feb 24;9(2):e89359. doi: 10.1371/journal.pone.0089359 (PMC3933416; doi:10.1371/journal.pone.0089359)

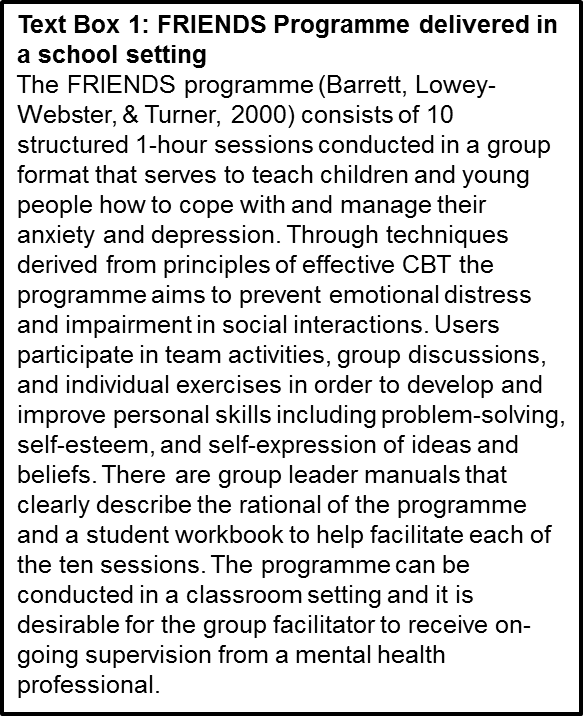

Supplement: Text Box S1 — FRIENDS programme delivered in a school setting. (TIF) [file pone.0089359.s003.tif]

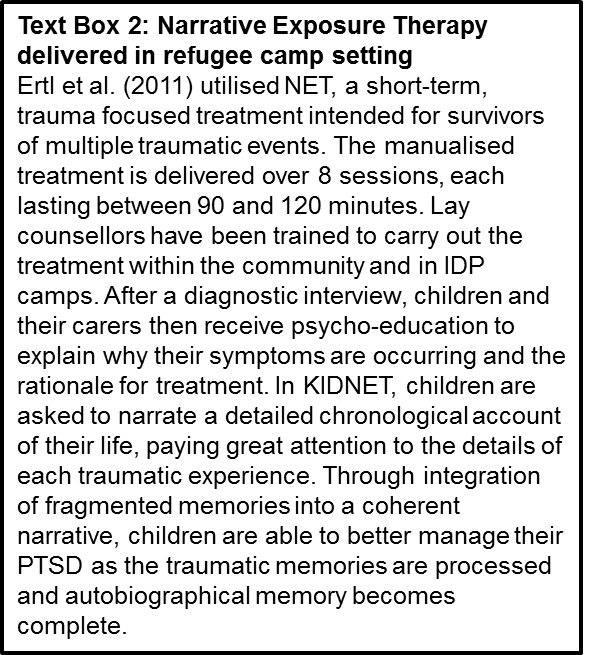

Supplement: Text Box S2 — Narrative Exposure Therapy delivered in refugee camp setting. (TIF) [file pone.0089359.s004.tif]
